# Isoform specific differences in phospholipase C beta 1 expression in the prefrontal cortex in schizophrenia and suicide

**DOI:** 10.1038/s41537-017-0020-x

**Published:** 2017-04-19

**Authors:** M. Udawela, E. Scarr, S. Boer, J. Y. Um, A. J. Hannan, C. McOmish, C. C. Felder, E. A. Thomas, B. Dean

**Affiliations:** 1 Molecular Psychiatry Laboratories, Florey Institute of Neuroscience and Mental Health, University of Melbourne, Parkville, VIC Australia; 2 Department of Psychiatry, University of Melbourne, Parkville, VIC Australia; 3 Cardiovascular and Neurology Products Division, Drug Evaluation Department, National Institute of Food and Drug Safety Evaluation, Osong Health Technology Administration Complex, Chungcheongbuk-do, South Korea; 4 Epigenetics and Neural Plasticity Laboratory, Florey Institute of Neuroscience and Mental Health, University of Melbourne, Parkville, VIC Australia; 5 Lilly Research Laboratories, Neuroscience Research Division, Eli Lilly and Company, Lilly Corporate Center, Indianapolis, IN USA; 6 Department of Molecular and Cellular Neuroscience, The Scripps Research Institute, La Jolla, CA USA

## Abstract

Our previous study demonstrated that phospholipase C beta 1 mRNA was down-regulated in Brodmann’s area 46 from subjects with schizophrenia. However, phospholipase C beta 1 protein has also been shown to be lower in Brodmann’s area 8 and 9 from teenage suicide subjects, creating a potential confound in interpreting the findings in schizophrenia due to the high suicide rate associated with this disorder. To begin to reconcile and consolidate these findings, in this study, we measured mRNA and protein levels of phospholipase C beta 1 variants a and b in Brodmann’s area 46 and Brodmann’s area 9 from subjects with schizophrenia, many of whom were suicide completers, and determined the diagnostic specificity of observed findings. Consistent with our previous study, levels of phospholipase C beta 1 a and b mRNA, but not protein, were lower in Brodmann’s area 46 from subjects with schizophrenia. In Brodmann’s area 9, phospholipase C beta 1a protein levels were lower in subjects with schizophrenia, while phospholipase C beta 1b mRNA was higher and protein was lower in those that had died of suicide. Altered protein levels in Brodmann’s area 9 appeared to be diagnostically specific, as we did not detect these changes in subjects with bipolar disorder, major depressive disorder or suicide completers with no diagnosis of mental illness. We further assessed the relationship between phospholipase C beta 1 and levels of muscarinic receptors (CHRMs) that signal through this protein, in both human and *Chrm* knockout mouse central nervous system tissue, and found no strong relationship between the two. Understanding central nervous system differences in downstream effector pathways in schizophrenia may lead to improved treatment strategies and help to identify those at risk of suicide.

## Suicide risk: Pinpointing protein variants

Variations in the expression of a specific protein may be linked to an increased risk of suicide in schizophrenia. The phospholipase C beta 1 (PLCB1) enzyme facilitates downstream signaling for multiple neurological systems, including dopamine regulation. Madhara Udewela at the Florey Institute, Australia, and co-workers measured post-mortem levels of two PLCB1 variants (a and b) in two areas of the prefrontal cortex (BA9 and BA46) in schizophrenia, many of whom were suicide completers. Levels of PLCB1a protein were lower in BA9 in patients with schizophrenia. In addition, PLCB1b protein levels were lower and PLCB1b RNA levels were higher in BA9 in those patients who died by suicide. However, these variations were not found in suicide victims who suffered from other mental health disorders. Changes in PLCB1 could provide a marker for suicide risk in schizophrenia.

## Introduction

Phospholipase C beta 1 (PLCB1) is a rate-limiting enzyme for downstream signalling of several neurotransmitter systems implicated in the pathophysiology of schizophrenia (Sz). Regulated by the Gq/11 family of GTP-binding proteins, PLCB1 facilitates signalling of dopamine via the D1 and D2 receptors,^1, 2^ serotonin via the 5-HT2A and 2C receptors,^3, 4^ glutamate via the group 1 metabotropic glutamate receptors^5, 6^ and acetylcholine via muscarinic receptors CHRM1, 3 and 5.^7^ Thus, PLCB1 may represent a point of convergence for a number of pathways known to be affected in Sz.^8–11^ In support of the argument that changes in PLCB1 could have a role in the pathophysiology, we,^12^ and others,^13, 14^ have reported differences in PLCB1 expression in the central nervous system (CNS) from subjects with Sz. In our previous study, we reported lower levels of *PLCB1* mRNA in Sz in Brodmann’s area (BA) 46 of the dorsolateral prefrontal cortex (DLPFC),^12^ a region largely implicated in cognitive deficits associated with the disorder. Thus these changes could in turn be affecting signalling through multiple neurotransmitter driven pathways. Importantly, lower PLCB1 protein levels and activity have been reported in another region of the DLPFC, BA8/BA9, from teenage suicide subjects,^15^ also suggesting that this protein represents a link between multiple systems that are disrupted in the brains of suicide victims. This is significant because Sz is associated with a high rate of suicide and therefore changes in PLCB1 levels may be a risk factor for suicide in people with Sz.

Supporting PLCB1’s involvement in Sz symptomatology, *Plcb1* knockout (^−/−^) mice show impairments in spatial memory, social behaviour and sensorimotor gating,^16, 17^ behaviours commonly considered to reflect symptomatology in patients with Sz. Interestingly, *Plcb1* ^−/−^ mice show reduced radioligand binding to CHRM1/4 in the cortex and hippocampus,^16^ again aligning with findings in subjects with Sz of decreased binding to and expression of these receptors in the same CNS regions.^18^ This interactive regulation between muscarinic receptors and PLCB1 displayed in the mice suggests that in subjects with Sz, disrupted muscarinic receptor levels in the CNS may be associated with reduced PLCB1 levels.

The CHRMs are of particular interest in the study of Sz due to their implications in cognitive deficits, which are now recognised as the most debilitating symptoms for patients to assimilate back into society and are still essentially resistant to available treatments.^19^ Prominently, we previously defined a subgroup of subjects with Sz, comprising around 26%, who showed a 74% reduction in radioligand binding to CHRM1 in BA9, which we have termed muscarinic receptor deficit Sz (MRDS), while the majority of subjects with Sz showed normal levels of binding compared to control.^20^ We have since shown that this deficit extends to other regions of the brain, and that MRDS subjects also show altered levels of CHRM1 promotor methylation, differential expression of various other genes,^21^ and decreased G-protein recruitment in response to CHRM1 orthosteric agonists.^22^ Given the importance of PLCB1 in muscarinic receptor signalling, we postulated that differences in the expression of this gene will be more pronounced in subjects with MRDS. In order to explore this hypothesis, we measured levels of PLCB1 mRNA and protein in post-mortem tissue of subjects with Sz, many of whom were suicide completers, from two DLPFC regions: BA46, where we first identified altered levels of PLCB1 in Sz, and BA9, where we first identified the MRDS subgroup, and which has been shown to display altered PLCB1 levels in suicide completers. This cohort consisted of subjects with MRDS, subjects with Sz having unchanged levels of CHRM1 in BA9 (non-MRDS), and matched non-psychiatric controls (cohort 1). To evaluate the diagnostic specificity of altered PLCB1 levels and the effect of suicide status, we also measured PLCB1 protein in BA9 from subjects with major depressive disorder (MDD), bipolar disorder (BD), suicide completers with no history of psychiatric illness, and matched non-psychiatric, non-suicide controls (cohort 2). BA24 was also assessed as we have recently shown lower levels of the serotonin 2A receptor in that region in subjects with mood disorders.^23^ Finally, PLCB1 levels were measured in the cortex of homozygous knockout (^−/−^) mice *Chrm1* ^−/−^, *Chrm2* ^−/−^, *Chrm4* ^−/−^ and *Chrm5* ^−/−^ to determine whether changes in levels of cortical muscarinic receptors could drive changes in PLCB1.

## Results

### Human demographic data

In cohort 1a (mRNA) and 1b (protein) there were no significant differences in the mean age (1a *p* = 0.98, 1b *p* = 0.88), brain pH (1a *p* = 0.06, 1b *p* = 0.10), post-mortem interval (PMI; 1a *p* = 0.49, 1b *p* = 0.50) or sex ratios (1a *p* = 0.09, 1b *p* = 0.10), and no difference in RNA integrity number (RIN) in cohort 1a (BA9 *p* = 0.27, BA46 *p* = 0.86), with diagnosis (Table 1). Experimental measures showed normal distribution and homogeneity of variance, and did not strongly correlate with any potential confounds (<0.01 < *r* ^2^ < 0.19; Supplementary Table S1). There was a significant difference in the number of people that died as a result of suicide in both cohort 1a (*p* = 0.0002) and 1b (*p* = 0.005) (Table 1), rendering this factor an irrevocable confound. Hence, experimental data were subsequently analysed comparing PLCB1 levels between suicide completers and those who died of other causes.

**Table 1:** Summary of demographic data for cohort 1 (Mean ± SEM)

	Control	Sz	Suicide	Non-suicide	MRDS	non-MRDS
(a) mRNA cohort	*n* = 26	*n* = 44	*n* = 16	*n* = 54	*n* = 20	*n* = 24
(b) protein cohort	*n* = 20	*n* = 38	*n* = 12	*n* = 46	*n* = 20	*n* = 18
Age (years)	45.7 ± 3.3	45.8 ± 2.5	30.8 ± 2.5	50.2 ± 2.1	46.1 ± 3.8	45.7 ± 3.4
	45.8 ± 3.8	46.5 ± 2.8	29.3 ± 2.7	50.6 ± 2.3	“	46.9 ± 4.1
Brain pH	6.32 ± 0.04	6.21 ± 0.04	6.26 ± 0.04	6.25 ± 0.06	6.25 ± 0.05	6.18 ± 0.06
	6.32 ± 0.05	6.21 ± 0.04	6.26 ± 0.03	6.25 ± 0.04	“	6.17 ± 0.06
PMI (hours)	42.9 ± 3.2	40.4 ± 2.0	44.0 ± 3.8	40.5 ± 1.9	39.0 ± 2.7	41.6 ± 3.0
	42.7 ± 3.4	40.2 ± 2.1	44.7 ± 4.0	40.3 ± 2.0	“	41.5 ± 3.3
Sex	22M/4F	36M/8F	15M/1F	43M/11F	16M/4F	20M/4F
	17M/3F	31M/7F	11M/1F	35M/11F	“	14M/4F
Suicide	26N	16Y/28N	16Y	54N	7Y/13N	9Y/15N
	20N	12Y/26N	12Y	46N	“	5Y/13N
DOI (years)	N/A	18.9 ± 2.2	7.81 ± 1.48	25.2 ± 2.7	19.3 ± 3.6	18.5 ± 2.8
		19.7 ± 2.5	6.75 ± 1.65	25.3 ± 2.9	“	19.7 ± 3.5
Antipsychotic drug dose (mg)^a^	0	629 ± 98	786 ± 201	294 ± 68	593 ± 124	661 ± 151
	0	598 ± 90	662 ± 178	340 ± 78	“	604 ± 135
Lifetime drug exposure (g)^a^	0	12.1 ± 2.3	6.00 ± 1.89	7.48 ± 1.99	13.2 ± 4.1	11.2 ± 2.7
	0	13.2 ± 2.7	5.57 ± 2.56	9.10 ± 2.30	“	13.2 ± 3.6
Anti-ach	26N	20Y/24N	8Y/8N	12Y/16N	11Y/9N	9Y/15N
	20N	19Y/19N	7Y/5N	12Y/14N	“	8Y/10N
RIN BA46	7.62 ± 0.16	7.65 ± 0.18	7.69 ± 0.31	7.63 ± 0.14	7.73 ± 0.23	7.56 ± 0.28
	N/A	N/A	N/A	N/A	N/A	N/A
RIN BA9	8.47 ± 0.18	8.10 ± 0.23	7.94 ± 0.41	8.33 ± 0.17	8.78 ± 0.14	7.54 ± 0.38
	N/A	N/A	N/A	N/A	N/A	N/A

*DOI* duration of illness, *F* female, *M* male, *MRDS* muscarinic receptor deficit schizophrenia, *N* no, *N/A* not applicable, *PMI* post-mortem interval, *Sz* schizophrenia, *Y* yes

^a^ Chlorpromazine equivalents

Dividing subjects based on suicide status showed no difference in pH (cohort 1a *p* = 0.83, 1b *p* = 0.84), PMI (1a *p* = 0.40, 1b *p* = 0.33), sex ratios (1a *p* = 0.27, 1b *p* = 0.43), or lifetime antipsychotic drug exposure (1a *p* = 0.63, 1b *p* = 0.47), and no difference in RIN in cohort 1a (BA9 *p* = 0.31, BA46 *p* = 0.82). There were significant differences in mean age (*p* < 0.0001 cohort 1a and b) and in duration of illness (DOI; 1a *p* < 0.0001, 1b *p* < 0.001), due to suicide subjects dying younger (Table 1). Final recorded antipsychotic dose also varied between suicide completers and those who died of other causes (1a *p* = 0.004, but not 1b *p* = 0.08), as controls receive no antipsychotic medication. There were no strong correlations, however, between these factors and PLCB1 levels (Supplementary Table S1) and they were therefore unlikely to affect the statistical outcomes of the study.

When subjects with Sz were separated into MRDS and non-MRDS, there were no differences in mean age (cohort 1a *p* = 0.10, 1b *p* = 0.99), brain pH (1a *p* = 0.10, 1b *p* = 0.15) PMI (1a *p* = 0.53, 1b *p* = 0.15) or sex ratios (1a *p* = 0.91, 1b *p* = 0.84) between the three groups when compared with control. Frequency of suicide was different across groups (Df_2_ = 12.29, *p* = 0.002, Df_2_ = 8.27, *p* = 0.02; Table 1) due to none of the control subjects dying by suicide. RIN values for BA9 tissue were different across the groups (Df_2_ = 5.96, *p* = 0.004; Table 1) but there was no strong correlation between this factor and experimental data (Supplementary Table S1). DOI (1a *p* = 0.88, 1b *p* = 0.93), average last recorded antipsychotic drug dose (1a *p* = 0.74, 1b *p* = 0.95), lifetime drug exposure (1a *p* = 0.68, 1b *p* = 1.00) and number of people that were prescribed anticholinergic drugs (1a *p* = 0.83, 1b *p* = 0.86) did not differ between MRDS and non-MRDS (Table 1).

In cohort 2 there were no significant differences in mean age (*p* = 0.48), PMI (*p* = 0.41), sex ratios (*p* = 0.43) or DOI (*p* = 0.62) between the groups (Table 2). There was significant variance in the rate of suicide (Df_3_ = 32.7, *p* < 0.0001) and pH (F_3,49_ = 6.50, *p* < 0.001), where the MDD group had higher pH than control and BD (*p* < 0.01, both; Table 2). Dividing this cohort based on suicide status showed a difference in age (*p* = 0.006) and pH (*p* = 0.0001) but no difference in DOI (*p* = 0.77) or PMI (*p* = 0.43; Table 2). The experimental data showed no strong correlation with any of the potential confounds (Supplementary Table S1), therefore these factors are unlikely to affect the outcomes. Experimental data was normally distributed and showed homogeneity of variance.

**Table 2:** Summary of demographic data for cohort 2 (Mean ± SEM)

	Control *n* = 14	MDD *n* = 15	BD *n* = 15	Suicide no Dx *n* = 9	Suicide *n* = 27	Non-suicide *n* = 26
Age (years)	60.3 ± 3.7	59.3 ± 4.2	59.4 ± 3.2	51.9 ± 3.1	53.1 ± 2.6	63.3 ± 2.4
Brain pH	6.26 ± 0.07	6.56 ± 0.04	6.26 ± 0.06	6.46 ± 0.07	6.50 ± 0.04	6.25 ± 0.05
PMI (hours)	45.2 ± 4.3	44.1 ± 4.0	38.0 ± 3.8	36.6 ± 4.7	39.7 ± 2.9	43.09 ± 3.1
Gender	7M/7F	7M/8F	7M/8F	7M/2F	16M/11F	12M/14F
Suicide	14N	13Y/2N	5Y/10N	9Y	27Y	26N
DOI (years)	N/A	16.3 ± 2.7	18.5 ± 3.5	N/A	17.2 ± 2.8	18.5 ± 3.4

*BD* bipolar disorder, *DOI* duration of illness, *Dx* diagnosis, *F* female, *M* male, *MDD* major depressive disorder, *N* no, *N/A* not applicable, *PMI* post-mortem interval, *Sz* schizophrenia, *Y* yes

### PLCB1 mRNA and protein levels in cohort 1

#### Brodmann’s area 46

**Fig. 1:**
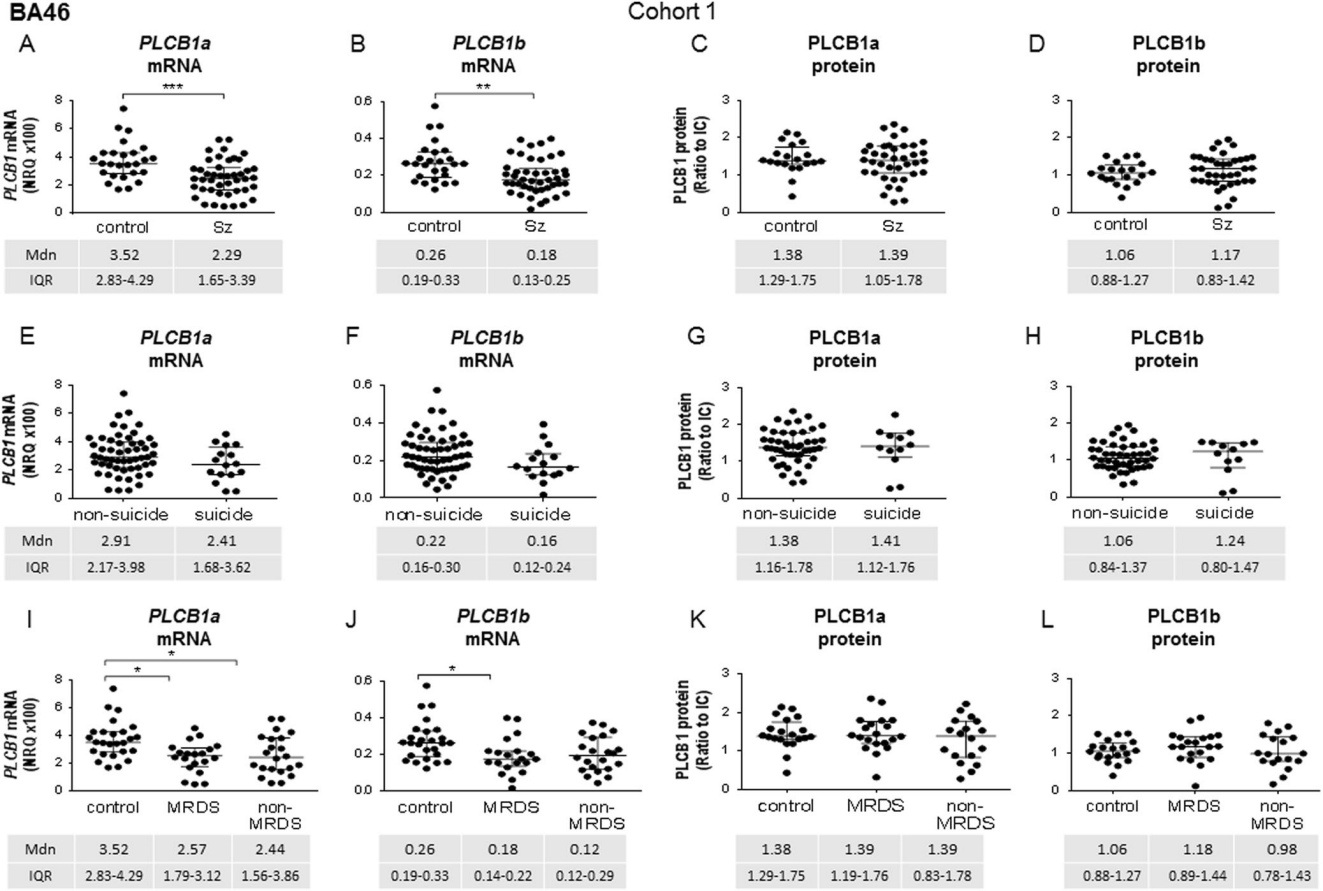
*PLCB1a* and *PLCB1b* mRNA (*left*) and PLCB1a and PLCB1b protein (*right*) levels in cohort 1 measured in tissue from BA46, analysed as Sz vs. control (**a**–**d**), as suicide completers vs. non-suicide (**e**–**h**), and with Sz subjects divided into MRDS and non-MRDS compared to control (**i**–**l**). *Error bars* show median (Mdn) and IQR

Levels of *PLCB1a* (*U* = 298, *p* < 0.001) and *b* (*U* = 325, *p* < 0.01) mRNA were lower in subjects with Sz compared to control in BA46 (Figs. 1a, b). By contrast, levels of PLCB1a (*U* = 329, *p* = 0.62) or PLCB1b (*U* = 345, *p* = 0.57) protein did not differ in BA46 from subjects with Sz compared to control (Fig. 1c, d).

When subjects in cohort 1 were separated based on suicide status, neither *PLCB1a* (*U* = 316, *p* = 0.11, Fig. 1i) and *PLCB1b* mRNA (*U* = 307, *p* = 0.08), nor PLCB1a and PLCB1b protein (*U* = 271, *p* = 0.93, U = 257, *p* = 0.73, respectively), showed significant variation between suicide completers and those who died of other causes (Fig. 1e–h).

When subjects with Sz were divided into MRDS and non-MRDS, and compared to controls, there was significant variance in levels of *PLCB1a* (*H* = 10.66, *p* = 0.005) and *PLCB1b* (*H* = 8.19, *p* = 0.017) mRNA. Post-hoc tests revealed that the variance in *PLCB1a* mRNA was due to lower levels in tissue from both groups of subjects with Sz compared to control (*p* = 0.01 MRDS, *p* = 0.03 non-MRDS, vs. control, Fig. 1i). By contrast, lower levels of *PLCB1b* mRNA were only detected in subjects with MRDS (*p* = 0.03, Fig. 1j). Levels of PLCB1a (*H* = 0.86, *p* = 0.65) and PLCB1b protein (*H* = 1.64, *p* = 0.49) did not differ between MRDS and non-MRDS compared to controls (Fig. 1k, l).

#### Brodmann’s area 9

**Fig. 2:**
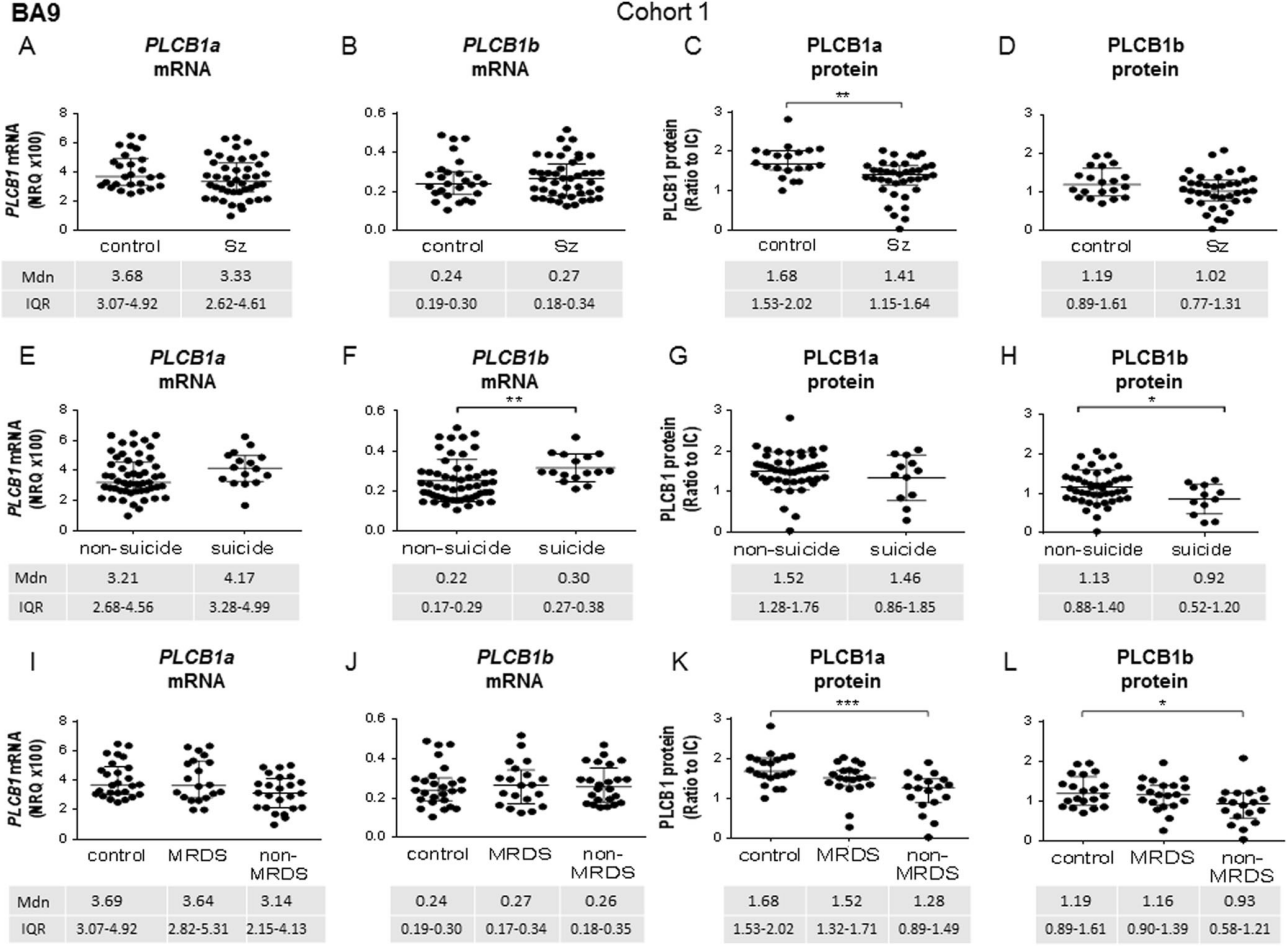
*PLCB1a* and *PLCB1b* mRNA (*left*), and PLCB1a and PLCB1b protein (*right*) levels in cohort 1 measured in tissue from BA9, analysed as Sz vs. control (**a**–**d**), as suicide completers vs. non-suicide (**e**–**h**), and with Sz subjects divided into MRDS and non-MRDS compared to control (**i**–**l**). Error bars show median (Mdn) and IQR

Levels of *PLCB1a* (*U* = 448, p = 0.13) and *PLCB1b* (*U* = 537, *p* = 0.67) mRNA did not differ between subjects with Sz and control in BA9 (Fig. 2a, b), while protein levels of PLCB1a (U = 189, *p* = 0.001, Fig. 2c), but not PLCB1b (U = 281, *p* = 0.11, Fig. 2d) were lower in subjects with Sz.

When these subjects were separated based on suicide status, levels of *PLCB1*a mRNA were not different (*U* = 301, *p = *0.14, Fig. 2e) while *PLCB1*b mRNA was higher in suicide (*U* = 131, *p* = 0.005, Fig. 2f) compared to non-suicide. At the level of protein, PLCB1a was not different (*U* = 241, *p = *0.50, Fig. 2g), while PLCB1b was lower in suicide completers (*U* = 170, *p* = 0.04, Fig. 2h). Removing the control subjects from this analysis to compare suicide status within Sz showed a significant difference in both *PLCB1a* and *b* mRNA but no difference in protein (see Supplementary Table S2).

When subjects with Sz were divided into MRDS and non-MRDS, there was no significant variance in *PLCB1a* (*H* = 5.43, *p* = 0.07), or *PLCB1b* (*H* = 0.18, *p* = 0.91; Fig. 2i and j). By contrast, levels of PLCB1a (*H* = 14.53, *p* < 0.001) and PLCB1b (*H* = 7.11, *p* = 0.03) protein varied, due to lower levels of both isoforms in non-MRDS compared to control (PLCB1a *p* < 0.001, PLCB1b *p* = 0.04, Fig. 2k, l).

### PLCB1 protein levels in cohort 2

#### Brodmann’s area 9

**Fig. 3:**
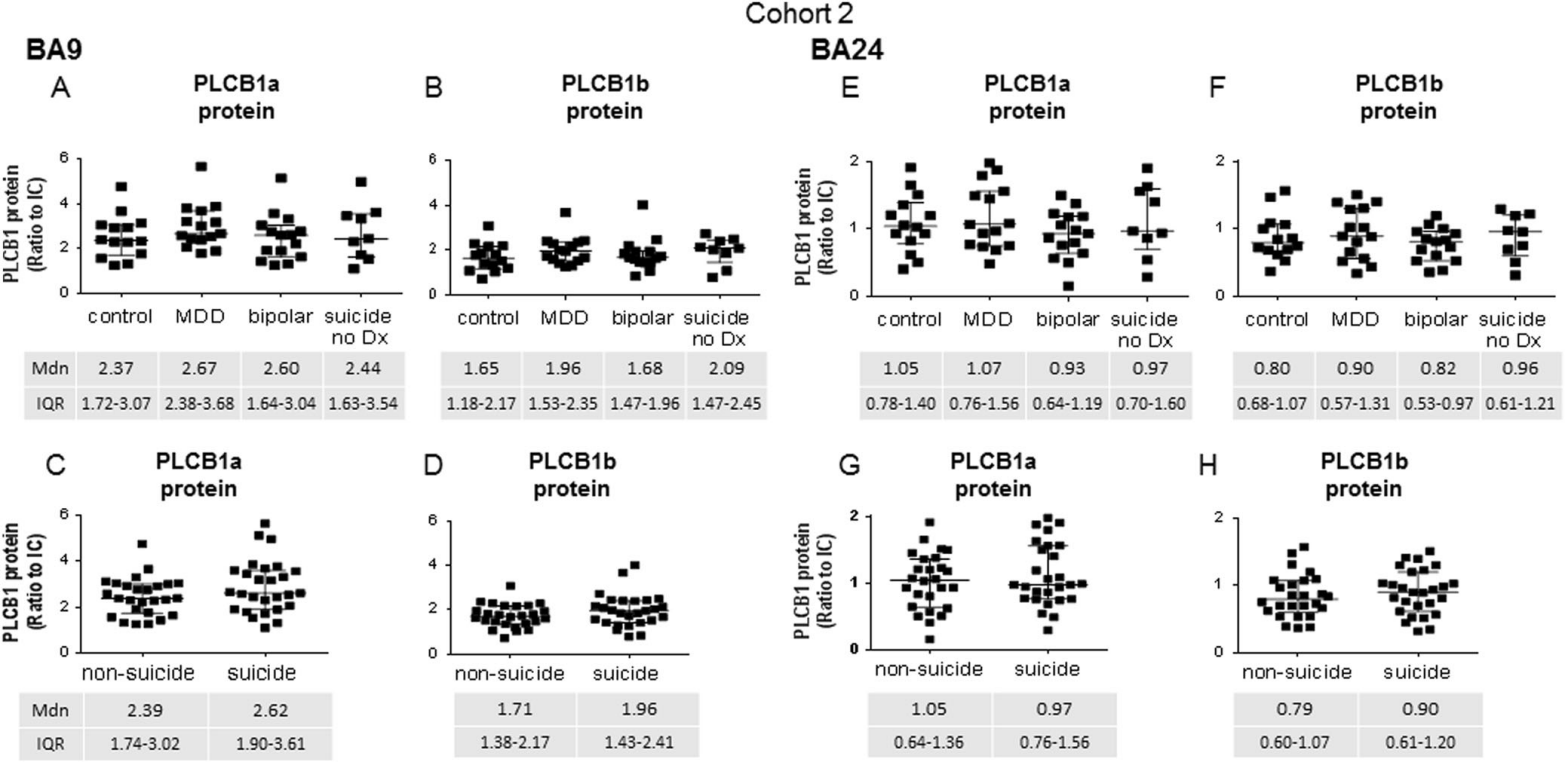
PLCB1a and PLCB1b protein levels in cohort 2 measured in tissue from BA9 (**a**–**d**) and BA26 (**e**–**h**), analysed as subjects with MDD, BD, and subjects who died of suicide who had no history of psychiatric illness (suicide no Dx), compared to control (**a**), (**b**), (**e**), (**f**), and with all subjects in this cohort divided into suicide completers vs. non-suicide (**c**), (**d**), (**g**), (**h**). *Error bars* show median (Mdn) and IQR

Comparing PLCB1 protein levels in subjects with MDD, BD, subjects who completed suicide without a history of psychiatric illness (suicide no Dx), and control, showed no variance in PLCB1a (*H* = 2.48, *p* = 0.48) or PLCB1b (*H* = 3.10, *p* = 0.38) across the groups in BA9 (Fig. 3a, b). Brain pH, which was found to be different between the groups, did not affect the outcome (PLCB1a *p* = 0.99, PLCB1b *p* = 0.83)

Analysing the data with these subjects grouped according to suicide status also showed no significant difference in PLCB1 protein levels (PLCB1a *U* = 269, *p* = 0.14, PLCB1b *U* = 276, *p* = 0.19, Fig. 3c, d). Analysing by suicide status within the diagnosis of MDD or BD also showed no significant difference in protein levels of either isoform (see Supplementary Table S2).

#### Brodmann’s area 24

There was no variance in levels of PLCB1a (*H* = 1.95, *p* = 0.58) or PLCB1b (*H* = 1.59, *p* = 0.66) protein between MDD, BD, suicide no Dx, and control in BA24 (Fig. 3e, f). Brain pH did not affect the outcome (PLCB1a *p* = 0.34, PLCB1b *p* = 0.41)

Analysing the data based on suicide status also showed no difference in levels (PLCB1a *U* = 321, *p* = 0.60, PLCB1b *U* = 313, *p* = 0.50, Fig. 3g, h).

### PLCB1 mRNA and protein levels in Chrm Knockout mice

#### Mouse cortex

**Fig. 4:**
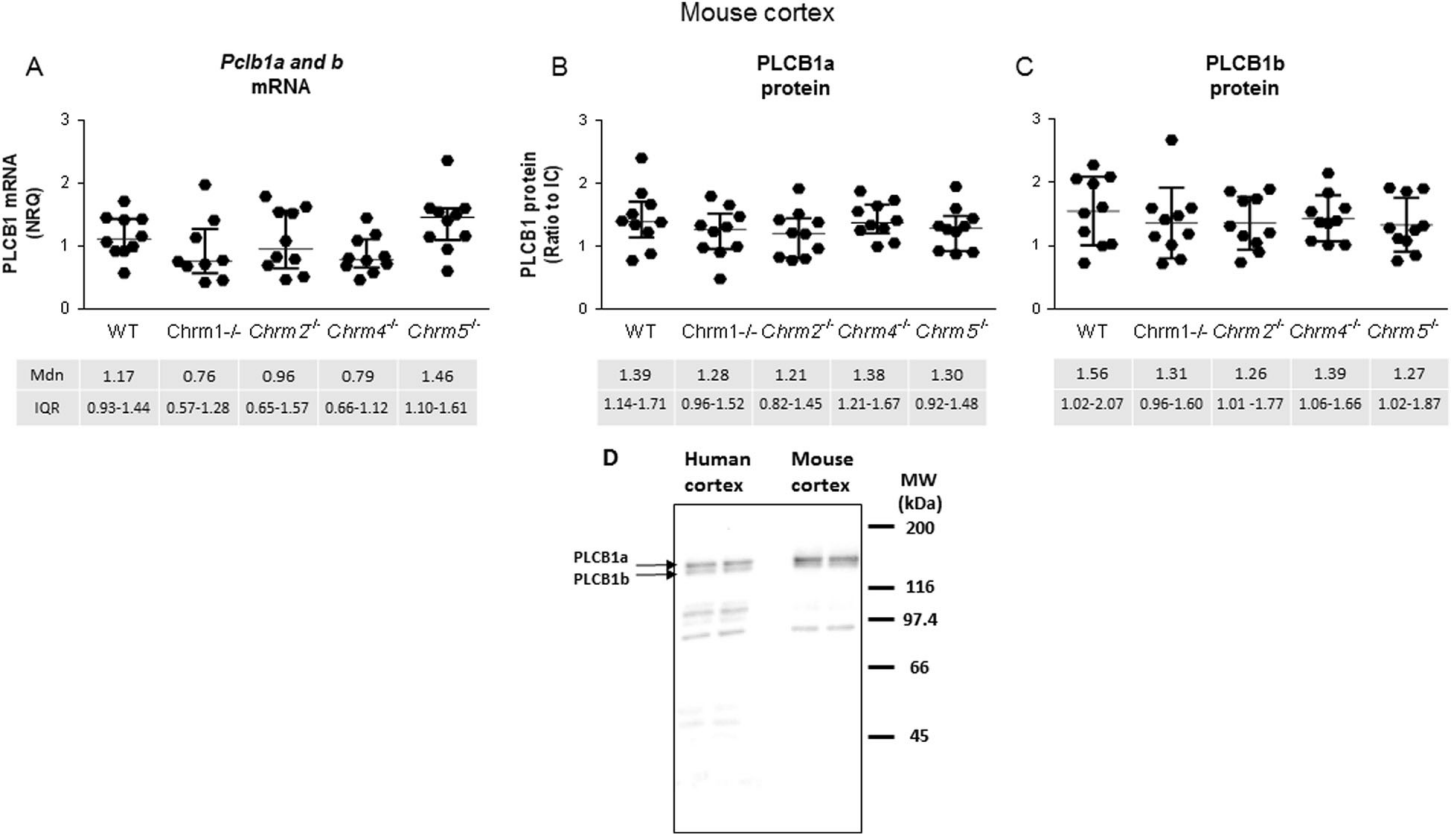
*PLCB1 a* and *b* mRNA (**a**), and PLCB1a (**b**) and PLCB1b (**c**) protein, levels measured in CNS from *Chrm1, 2, 4* and *5* knockout (^−/−^) mice compared to WT mice. *Error bars* show median (Mdn) and IQR. Western blot image (**d**) of 5 ug human or mouse cortical homogenate, run in duplicate, probed with anti-PLCB1 antibody

Experimental data showed normal distribution and homogeneity of variance. There was no significant difference in levels of *Plcb1* mRNA in the cortex of *Chrm1* ^−/−^, *Chrm2* ^−/−^, *Chrm4* ^−/−^, *Chrm5* ^−/−^ and wild-type (WT) mice (*H* = 8.81, *p* = 0.07; Fig. 4a). Protein levels also showed no variance in either PLCB1a (*H* = 3.35, *p* = 0.50) or PLCB1b (*H* = 1.40, *p* = 0.85) with genotype (Fig. 4b and c).

## Discussion

This study describes complex changes in PLCB1 in the cortex of subjects with Sz, and in those who were suicide completers. By measuring each isoform of PLCB1 we were able to discern isoform specific variations. Consistent with our previous report,^12^ levels of PLCB1 a and b mRNA were lower in BA46 from subjects with Sz, however protein levels were not different. By contrast, while levels of PLCB1 mRNA did not vary in BA9, protein levels of PLCB1a were lower in this cortical region in subjects with Sz. In this study we could not match suicide rates between subjects with Sz and controls, and therefore analysed our data comparing all suicide completers to those who died of other causes. This analysis revealed higher levels of PLCB1b mRNA and lower levels of PLCB1b protein in BA9 from subjects with Sz who died of suicide, compared to non-suicide in that cohort, suggesting an up-regulation of PLCB1b gene expression as a compensatory response to low levels of protein in the cortex of suicide completers. Additionally, our data reflect isoform specific differences in Sz and suicide completers. Despite their high sequence homology these two isoforms exhibit varying expression levels between tissue, cell compartments, and with age, and there is evidence that responses initiated by α_1_-adrenergic receptor activation in cardiomyocites involves only isoform b,^24^ indicating they have distinct physiological functions. Thus, isoform expression differences across brain regions and cell compartments may reflect distinct regulatory mechanisms.^25^ Our findings displayed diagnostic specificity, with no differences in PLCB1 detected in BA9 from subjects with MDD, BD, or suicide with no history of psychiatric illness. We also report no variance in PLCB1 protein levels in BA24 in these subjects, a region previously shown to be impacted in MDD.^23^ Abnormalities in PLCB1 levels could therefore play a role in Sz pathophysiology, and represent a biological marker for Sz as well as those with the disorder who are at risk of suicide dependent on the isoform measured.

Our findings do not agree with one study that reported higher levels of PLCB1 protein in BA9 from people with Sz.^13^ Our cohort had higher PMI values (42 h) compared to the previous study (10 h^13^), which could possibly affect protein measures, accounting for the different outcomes, however our pH and RIN values indicate good tissue preservation and quality, thus further experiments would need to be carried out to confirm the effects of PMI on PLCB1 measures. Another possible explanation for these divergent results is that the previous study measured both isoforms together, and detected altered PLCB1 levels in insoluble tissue fraction only, whereas our measures were of each isoform, from total tissue homogenate, suggesting there may be isoform and cell compartment specific changes in PLCB1 in the CNS of people with Sz, reflecting complex regulation of this protein. The same study reported lower levels of PLCB1 in the left but not right superior temporal cortex of subjects with Sz and unchanged levels of the protein in the nucleus accumbens and amygdala,^13^ supporting our findings of CNS regional selectivity. While the altered protein levels observed in BA9 is expected to have a functional outcome, the significance of altered gene expression in the absence of a change in protein levels as observed in BA46 is difficult to interpret. Recent evidence that local protein synthesis can occur rapidly in neurons in response to stimuli^26^ would suggest that altered mRNA levels can affect a cells ability to swiftly modify synaptic transmission. The diagnostic specificity of our findings aligns with studies reporting a PLCB1 gene deletion in 5 out of 15 people with Sz,^27^ while only occurring in one out of 15 BD patients,^28^ and in none of the 15 MDD patients examined.^29^ While there are reports of other PLC isoforms being associated with BD pathogenesis and treatment response,^30, 31^ our findings are consistent with PLCB1 being not highly implicated in affective disorders.^27^

Our data from cohort 1 analysed by suicide status is consistent with the earlier finding of lower PLCB1 protein in membrane and cytosol fractions from BA8/9 in adolescent suicide regardless of history of mental illness,^15, 32^ however this is not supported by our findings in cohort 2 of no difference in suicide completers with MDD, BD or no history of mental illness. These data are consistent, however, with another study that showed no difference in PLCB1 protein levels in BA8/9 from suicide completers with MDD compared to non-psychiatric controls that died of other causes.^33^ These differences may be explained by the age of the subjects; the study of adolescent suicide and our cohort 1, where altered levels of PLCB1 were seen in the suicide brain, consisted of younger suicide subjects (16 ± 2 years^15^ and 29 ± 3 years, respectively), compared to the study of suicide in MDD (59 ± 4 years^33^) and our cohort 2 (53 ± 2 years), where PLCB1 levels were not altered. In line with this, a recent study showed that loss of PLCB1 in the brains of suicide completers occurred only in younger subjects aged less than 29 years.^34^ Thus is it possible that lower PLCB1b protein levels in BA9 could be a marker of suicide only in younger populations.

In designing our study we postulated that markedly low levels of CHRM1 in the cortex of a subset of subjects with Sz may impact PLCB1 in those individuals. In BA46, lower levels of PLCB1 mRNA were seen in both sub-groups of subjects with Sz regardless of muscarinic receptor levels; PLCB1 protein remained not different in BA46. In BA9, differences in PLCB1 protein levels were specific to the non-MRDS subjects. Hence these data largely suggest that lower levels of muscarinic M1 receptors in the cortex did not influence levels of PLCB1. Moreover, these data indicate that our previous findings of differences in oxotremorine-M-induced Gαq/11-[35^S^]-GTPγS binding in BA9 from MRDS^22^ are not due to altered feedback from PLCB1. In addition, we show that neither PLCB1 mRNA nor protein was different in *Chrm1* ^−/−^ mice, however these data need to be interpreted with caution due to the WT and knockout mice being obtained from a different sources, and thus further studies would be needed to confirm this finding. Combined with previous findings of low [H^3^]-PZP binding in *Plcb1* ^−/−^ mice,^16^ it would seem that while changes in levels of PLCB1 may affect levels of CHRM1, the reverse relationship is not apparent.

As with all research into psychiatric disorders, the effect of treatment is a potential confound. It has been shown that, while 21 days administration of clozapine or haloperidol had no effect, chlorpromazine treatment decreased PLCB1 activity and expression in the rat cortex, hippocampus, cerebellum and striatum.^35^ However, we saw no correlation between antipsychotic drug dose and PLCB1 mRNA or protein levels in subjects with Sz, minimising the likelihood that treatment was affecting the outcomes of our study. Treatment with antidepressants has also been shown to decrease levels of PLCB1 activity and expression in the rat cortex and hippocampus,^36^ yet we saw no difference in PLCB1 levels in CNS tissue from subjects with MDD or BD, many of whom were on antidepressant treatment regimes, suggesting that the rat and human CNS may respond differently to antipsychotic and antidepressant treatments. Furthermore, the divergent findings for mRNA and protein observed in the human tissue suggest there may be more complex regulation of this protein in human CNS compared to rodent CNS. Taken together with studies that show rat lines that have differing levels of PLCB1 in their CNS respond differently to antidepressant treatments,^37, 38^ those studies do suggest that PLCB1 may be a central target of these drugs, and therefore CNS levels of this protein may be an important factor in treatment response.

Together, our findings suggest that changes in PLCB1a may contribute to the pathophysiology of Sz. This supports findings in *Plcb1* ^−/−^ mice showing that loss of this protein leads to abnormalities characteristic of Sz.^16, 17^ In addition, altered levels in expression and activity of Plcb1 in the rat hippocampus and cortex have been shown to influence learning and behaviour.^39^ *Plcb1* ^−/−^ mice also display other aspects of face validity as an animal model of Sz, including cortical maldevelopment^6, 40^ and hippocampal dysfunction,^16, 41^ as well as predictive validity.^16^ Furthermore, genetic linkage studies mapped a Sz susceptibility locus near the region encoding the *PLCB1* gene.^42, 43^ As this protein is a downstream signalling protein for all receptors that signal through Gq/11, a number of systems would be affected by a change in PLCB1 levels, warranting further efforts towards understanding the regulation of this protein in the CNS and its implications in CNS disorders and treatment.

In summary, we report PLCB1a protein is lower in BA9, but not BA46, from people with Sz, while PLCB1b protein is lower and mRNA is higher in those with the disorder that died of suicide, variances that were not observed among subjects with affective disorders or that died of suicide with no previous history of mental illness. Thus, further efforts are warranted to understand the role of PLCB1 in the pathophysiology of Sz, and to determine whether changes in PLCB1 are linked to an altered risk of suicide in people with the disorder and could therefore be a marker for suicide risk.

## Methods and materials

### Human post-mortem tissue collection

Consent to collect the CNS tissue that was used in this study was obtained from the Ethics Committee of the Victorian Institute of Forensic Medicine. Tissues were received from the Victorian Brain Bank, supported by The Florey Institute of Neuroscience and Mental

Health, The Alfred and the Victorian Forensic Institute of Medicine and funded in part by Parkinson’s Victoria and MND Victoriaand Mental Health. Informed consent for each tissue collection was obtained from the donor or senior next of kin. Psychiatric diagnoses were made as previously described.^20^ Suicide completion was accepted when listed as the cause of death by the Coroner. During the history review, information was gathered to allow the calculation of postmortem interval (PMI) as, when a death was witnessed, the time between death and autopsy. When death was not witnessed tissue was only collected from subjects who had been seen alive up to 5 h before being found dead. In those circumstances PMI was calculated as the time from being found to autopsy plus half of the time between last seen alive and being found dead. All cadavers were refrigerated within 5 h of being found dead. DOI was calculated as the time from first presentation to a psychiatric service to death, the final recorded antipsychotic drug dose was converted to mg chlorpromazine equivalents per day^44, 45^ and lifetime exposure was calculated as cumulative antipsychotic drug dose (in chlorpromazine equivalents) multiplied by years on each dose. All subjects were coded to remove subject identities. See Supplementary Table S3 for patient details.

It has become clear that PMI is not a clear indicator of preservation of CNS tissue for molecular studies, with CNS pH now being acknowledged as a better indicator of the overall quality of tissue.^46^ Hence, CNS pH was measured for each case as described previously.^47^ In addition, when mRNA was to be measured, RIN was measured as an indicator of the overall preservation of RNA in the CNS^46^ using an Agilent 2100 bioanalyser (Agilent Technologies, Santa Clara, CA, USA).

For this study tissue was obtained from 44 subjects with Sz and 26 control subjects who had no history of psychiatric illness that were matched as closely as possible for age, sex, postmortem interval (PMI) and brain pH (cohort 1a; Table 1 and Supplementary Table S2). The subjects with Sz consisted of 24 MRDS (defined by [^3^H]pirenzepine binding in BA9 of < 99 fmol/mg estimated tissue equivalence (ETE)) and 20 non-MRDS (defined by [^3^H]pirenzepine binding in BA9 of >102 fmol/mg ETE subjects^20^) subjects. Because of careful matching both the MRDS and non-MRDS groups matched closely to the controls with regards to age, sex, PMI and brain pH (Table 1). Due to limited tissue availability, PLCB1 protein was measured in sub-cohorts of these subjects being made up of 38 subjects with Sz (20 MRDS and 18 non-MRDS) and 20 controls (cohort 1b; Table 1). In addition, tissue was taken from 15 subjects with MDD, 15 subjects with BD, 9 subjects who had died of suicide with no history of psychiatric illness (suicide no Dx) and 14 control subjects (cohort 2; Table 2). To be able to match controls to these cases, a different cohort of controls had to be used (Supplementary Table S3). Our previous studies indicate cohorts of these sizes allow mean differences of ~15% to be reliably detected. Investigators were kept blinded to diagnosis allocations during sample preparation and subsequent experimentation.

All tissue for this study was from the left hemisphere. For studies in Sz and mood disorders tissue was excised from BA9 (lateral surface of the frontal lobe, including the middle frontal gyrus superior to the inferior frontal sulcus). In addition, for continuity with our previous studies,^12, 23^ for Sz studies tissue was also excised from BA46 (lateral surface of the frontal lobe and includes approximately the middle third of the middle frontal gyrus and the most rostral portion of the inferior frontal gyrus), and for mood disorders studies tissue was also excised from BA24 (ventral anterior cingulate gyrus around the genu of the corpus callosum).

### Animals

*Chrm* ^−/−^ mouse tissue was a generous gift from Lilly Research Laboratories (Indiana, IN, USA) from mice bred under contract by Taconic (Indiana, IN, USA).^48–51^ WT mice of the same background (C57BL/6N) were obtained from Monash Animal Services (Clayton, VIC, Australia). Whole brain tissue was collected from10 adult males each of *Chrm1* ^−/−^, *Chrm2* ^−/−^, *Chrm4* ^−/−^, *Chrm5* ^−/−^ and C57BL/6N following euthanasia by cervical dislocation. *Chrm3* ^−/−^ mice were not available to us at the time of experimentation. We have previously shown that animal cohorts of this size allow significant differences in CNS expression of 11% or greater to be readily identified. Excised tissue was rapidly frozen to −70 °C immediately following dissection. Coronal sections of about 2 mm thickness were taken from frozen tissue immediately posterior to Bregma for mRNA studies and the adjacent section was used for protein studies. Investigators were kept blinded to genotype during sample preparation and experimentation.

### RNA purification and first-strand cDNA synthesis

Total RNA was isolated from approximately 100 mg frozen tissue samples with 1.0 ml TRIzol^®^ reagent (Life Technologies, CA, USA). RNA from human tissue was extracted according to the manufacturer’s instructions, while mouse RNA was purified using the RNeasy RNA extraction kit (Qiagen, Limburg, Netherlands). All RNA was DNAse treated and tested for genomic DNA contamination, and RNA integrity was analysed on an Agilent 2100 bioanalyser (Agilent Technologies, CA, USA). cDNA was synthesised as described previously.^12^

### qPCR assay

cDNA was used as a template for qPCR, performed as previously described.^12^ Human *PLCB1* primers were designed using Beacon design software (Premier Biosoft International, CA, USA) to distinguish between the two *PLCB1* variants (*PLCB1a* forward 5′-ctggatgaaaagcccaagctg-3′, reverse 5′-attgctgtcttcactgatctttcct-3′*, PLCB1b* forward 5′-ggaaggttcctcctcattcttgt-3′, reverse 5′-cggaaggacggtggtcac-3′), with relative quantities normalising to the reference gene peptidylprolyl isomerase A (*PPIA*; forward primer 5′-atggtcaaccccaccgtgttcttcg-3′, reverse cgtgtgaagtcaccaccctgacaca-3′), which showed no variance between analysis groups. High sequence similarity between mouse *Pclb1* variants rendered it unfeasible to design primers that could distinguish the two. Therefore mouse *Pclb1* data is a measure of both variants (forward primer 5'-gcccctggagattctggagt-3′, reverse gggagacttgaggttcaccttt-3′). Relative quantities of mouse *Pclb1* were normalised to the reference gene mitochondrial methionyl-tRNA formyltransferase (*Mtfmt*), measured using primers sourced from PrimerBank^52^ (forward 5′-gaaaaatctgcccgaaagtctga-3′, reverse 5′-atggcaaggtgaagtctgagt-3′), which showed no variance between the genotypes. All amplicons were confirmed by sequencing prior to experimentation.

### Western blotting

Western blotting was performed on protein homogenate prepared from BA9, BA46 and BA24 and mouse CNS as previously described.^12^ Briefly, five ug of total protein was run on gels in duplicate for each sample. After transfer, equal protein loading was checked by Ponceau S staining of nitrocellulose membranes. Bands were then detected using mouse anti-PLCB1 antibody (Cat #610924, BD Biosciences) followed by HRP conjugated goat anti-mouse IgG (Cat #554002, BD Biosciences). The intensities of the 150 kDa and the 140 kDa bands (previously shown to correspond to PLCB1a and PLCB1b, respectively^24^) were measured in each sample (Fig. 4d). The antibody specificity was previously confirmed by the absence of these bands in *Plcb1* ^−/−^ mice.^12^ To control for inter-blot variation, an internal control (IC) sample, prepared from cerebellum tissue that was not part of the cohorts used, was run in 12 wells on two gels to establish both intra—and inter-blot variation for PLCB1 levels. This IC sample was included in duplicate on every gel and gels were imaged so that the optical density of this sample fell within the mean ± 1 SD obtained from the initial two gels. The density of PLCB1 in each sample was then expressed as a ratio to the IC.

### Statistical analysis

Demographic data were compared across groups, using a two-tailed *t* test or one-way analysis of variance for continuous variables, which show low sensitivity to deviations from normality, and *χ* ^2^ or Fisher’s exact tests for non-continuous variables. Linear regression was used to determine correlations between experimental data and continuous potential confounds, independent of diagnosis. Due to relatively small cohort sizes, *r* ^2^ > 0.49 was taken to indicate strong relationships between parameters.^53^ Distribution of the experimental data was assessed by KS normality and homogeneity of variance was assessed using F and Bartlett’s tests. Variance in experimental data with diagnoses, suicide status or genotype were analysed with non-parametric tests, due to the derivation of data as a ratio,^54^ using Mann–Whitney *U* test for comparing two groups or Kruskal–Wallis test for comparing more than two groups, which do not assume homogeneity of variance, followed by Dunn’s multiple comparison test to compare across groups for human tissue or against WT for animal tissue studies. The effect of pH on data from cohort 2 was analysed using analysis of covariance. All analyses were performed using GraphPad Prism v5.01 (GraphPad Software Inc., CA, USA). Experimental data are represented as median with interquartile range (IQR). Where multiple comparison tests were performed multiplicity adjusted *p* values are reported.

## Electronic supplementary material

Supplementary Table S1

Supplementary Figure S1

Supplementary Table S2

Supplementary Table S3

## References

[1] Lee SP (2004). Dopamine D1 and D2 receptor co-activation generates a novel phospholipase C-mediated calcium signal. J. Biol. Chem..

[2] Rashid AJ (2007). D1-D2 dopamine receptor heterooligomers with unique pharmacology are coupled to rapid activation of Gq/11 in the striatum. Proc. Natl. Acad. Sci. USA.

[3] Hagberg GB, Blomstrand F, Nilsson M, Tamir H, Hansson E (1998). Stimulation of 5-HT2A receptors on astrocytes in primary culture opens voltage-independent Ca2+channels. Neurochem. Int..

[4] Chang M, Zhang L, Tam JP, Sanders-Bush E (2000). Dissecting G protein-coupled receptor signaling pathways with membrane-permeable blocking peptides. Endogenous 5-HT(2C) receptors in choroid plexus epithelial cells. J. Biol. Chem..

[5] Conn PJ, Pin JP (1997). Pharmacology and functions of metabotropic glutamate receptors. Annu. Rev. Pharmacol. Toxicol..

[6] Hannan AJ (2001). PLC-beta1, activated via mGluRs, mediates activity-dependent differentiation in cerebral cortex. Nat. Neurosci..

[7] Kim D (1997). Phospholipase C isozymes selectively couple to specific neurotransmitter receptors. Nature.

[8] Howes OD, Kapur S (2009). The dopamine hypothesis of schizophrenia: version III--the final common pathway. Schizophr. Bull..

[9] Lieberman JA (1998). Serotonergic basis of antipsychotic drug effects in schizophrenia. Biol. Psychiatry.

[10] Konradi C, Heckers S (2003). Molecular aspects of glutamate dysregulation: implications for schizophrenia and its treatment. Pharmacol. Ther..

[11] Raedler TJ, Bymaster FP, Tandon R, Copolov D, Dean B (2007). Towards a muscarinic hypothesis of schizophrenia. Mol. Psychiatry.

[12] Udawela M, Scarr E, Hannan AJ, Thomas EA, Dean B (2011). Phospholipase C beta 1 expression in the dorsolateral prefrontal cortex from patients with schizophrenia at different stages of illness. Aust. N. Z. J. Psychiatry.

[13] Lin XH, Kitamura N, Hashimoto T, Shirakawa O, Maeda K (1999). Opposite changes in phosphoinositide-specific phospholipase C immunoreactivity in the left prefrontal and superior temporal cortex of patients with chronic schizophrenia. Biol. Psychiatry.

[14] Shirakawa O, Kitamura N, Lin XH, Hashimoto T, Maeda K (2001). Abnormal neurochemical asymmetry in the temporal lobe of schizophrenia. Prog. Neuropsychopharmacol. Biol. Psychiatry.

[15] Pandey GN (1999). Low phosphoinositide-specific phospholipase C activity and expression of phospholipase C beta1 protein in the prefrontal cortex of teenage suicide subjects. Am. J. Psychiatry..

[16] McOmish CE (2008). Phospholipase C-beta1 knockout mice exhibit endophenotypes modeling schizophrenia which are rescued by environmental enrichment and clozapine administration. Mol. Psychiatry.

[17] Koh HY, Kim D, Lee J, Lee S, Shin HS (2008). Deficits in social behavior and sensorimotor gating in mice lacking phospholipase Cbeta1. Genes Brain Behav..

[18] Dean B, Scarr E (2015). Possible involvement of muscarinic receptors in psychiatric disorders: a focus on schizophrenia and mood disorders. Curr. Mol. Med..

[19] Money TT (2010). Treating schizophrenia: novel targets for the cholinergic system. CNS Neurol. Disord. Drug Targets.

[20] Scarr E (2009). Decreased cortical muscarinic receptors define a subgroup of subjects with schizophrenia. Mol. Psychiatry.

[21] Scarr, E., Udawela, M., Thomas, E. A. & Dean, B. Changed gene expression in subjects with schizophrenia and low cortical muscarinic M1 receptors predicts disrupted upstream pathways interacting with that receptor. *Mol. Psychiatry*10.1038/mp.2016.195 (2016). 10.1038/mp.2016.195 PMC5794886 27801890

[22] Salah-Uddin H (2009). Altered M(1) muscarinic acetylcholine receptor (CHRM1)-Galpha(q/11) coupling in a schizophrenia endophenotype. Neuropsychopharmacology.

[23] Dean B (2014). Lower cortical serotonin 2A receptors in major depressive disorder, suicide and in rats after administration of imipramine. Int. J. Neuropsychopharmacol..

[24] Grubb DR, Vasilevski O, Huynh H, Woodcock EA (2008). The extreme C-terminal region of phospholipase Cbeta1 determines subcellular localization and function; the “b” splice variant mediates alpha1-adrenergic receptor responses in cardiomyocytes. FASEB J..

[25] Litosch I (2002). Novel mechanisms for feedback regulation of phospholipase C-beta activity. IUBMB Life.

[26] Huber KM, Kayser MS, Bear MF (2000). Role for rapid dendritic protein synthesis in hippocampal mGluR-dependent long-term depression. Science.

[27] Lo Vasco VR, Cardinale G, Polonia P (2012). Deletion of PLCB1 gene in schizophrenia-affected patients. J. Cell. Mol. Med..

[28] Lo Vasco VR, Longo L, Polonia P (2013). Phosphoinositide-specific Phospholipase C beta1 gene deletion in bipolar disorder affected patient. J. Cell. Commun. Signal..

[29] Lo Vasco VR, Polonia P (2012). Molecular cytogenetic interphase analysis of phosphoinositide-specific phospholipase C beta1 gene in paraffin-embedded brain samples of major depression patients. J. Affect. Disord..

[30] Turecki G (1998). Evidence for a role of phospholipase C-gamma1 in the pathogenesis of bipolar disorder. Mol. Psychiatry.

[31] Dawson E (1995). Genetic association between alleles of pancreatic phospholipase A2 gene and bipolar affective disorder. Psychiatr. Genet..

[32] Pandey, G. N. in *Risk factors for suicide: summary of a workshop* (ed. Insitute of medicine) 10–11 (The National Academies Press, 2001). 25057580

[33] Pacheco MA (1996). Alterations in phosphoinositide signaling and G-protein levels in depressed suicide brain. Brain Res..

[34] Lo Vasco VR (2015). Impairment and reorganization of the phosphoinositide-specific phospholipase C enzymes in suicide brains. J. Affect. Disord..

[35] Dwivedi Y, Pandey GN (1999). Effects of treatment with haloperidol, chlorpromazine, and clozapine on protein kinase C (PKC) and phosphoinositide-specific phospholipase C (PI-PLC) activity and on mRNA and protein expression of PKC and PLC isozymes in rat brain. J. Pharmacol. Exp. Ther..

[36] Dwivedi Y, Agrawal AK, Rizavi HS, Pandey GN (2002). Antidepressants reduce phosphoinositide-specific phospholipase C (PI-PLC) activity and the mRNA and protein expression of selective PLC beta 1 isozyme in rat brain. Neuropharmacology.

[37] Piras G, Piludu MA, Giorgi O, Corda MG (2014). Effects of chronic antidepressant treatments in a putative genetic model of vulnerability (Roman low-avoidance rats) and resistance (Roman high-avoidance rats) to stress-induced depression. Psychopharmacology (Berl)..

[38] Piras G, Giorgi O, Corda MG (2010). Effects of antidepressants on the performance in the forced swim test of two psychogenetically selected lines of rats that differ in coping strategies to aversive conditions. Psychopharmacology (Berl.).

[39] Salles J (2001). Transmembrane signaling through phospholipase C in cortical and hippocampal membranes of psychogenetically selected rat lines. Psychopharmacology (Berl.).

[40] Spires TL (2005). Activity-dependent regulation of synapse and dendritic spine morphology in developing barrel cortex requires phospholipase C-beta1 signalling. Cereb. Cortex.

[41] Manning EE, Ransome MI, Burrows EL, Hannan AJ (2012). Increased adult hippocampal neurogenesis and abnormal migration of adult-born granule neurons is associated with hippocampal-specific cognitive deficits in phospholipase C-beta1 knockout mice. Hippocampus.

[42] Arinami T (2005). Genomewide high-density SNP linkage analysis of 236 Japanese families supports the existence of schizophrenia susceptibility loci on chromosomes 1p, 14q, and 20p. Am. J. Hum. Genet..

[43] Fanous AH (2008). Novel linkage to chromosome 20p using latent classes of psychotic illness in 270 Irish high-density families. Biol. Psychiatry.

[44] Woods SW (2003). Chlorpromazine equivalent doses for the newer atypical antipsychotics. J. Clin. Psychiatry.

[45] Remington, G. J. in *Clincal Handbook of Psychotropic Drugs* 15th Rev edn, (eds Bezchlibnyk-Butler K. Z., Jeffries J. J., & Procyshyn R. M.) (Hogrefe, 1999).

[46] Stan AD (2006). Human postmortem tissue: what quality markers matter?. Brain Res..

[47] Kingsbury AE (1995). Tissue pH as an indicator of mRNA preservation in human post-mortem brain. Brain Res. Mol. Brain Res..

[48] Gomeza J (1999). Pronounced pharmacologic deficits in M2 muscarinic acetylcholine receptor knockout mice. Proc. Natl. Acad. Sci. USA.

[49] Gomeza J (1999). Enhancement of D1 dopamine receptor-mediated locomotor stimulation in M(4) muscarinic acetylcholine receptor knockout mice. Proc. Natl. Acad. Sci. USA.

[50] Porter AC (2002). M1 muscarinic receptor signaling in mouse hippocampus and cortex. Brain Res..

[51] Yamada M (2001). Cholinergic dilation of cerebral blood vessels is abolished in M(5) muscarinic acetylcholine receptor knockout mice. Proc. Natl. Acad. Sci. USA.

[52] Spandidos A, Wang X, Wang H, Seed B (2010). PrimerBank: a resource of human and mouse PCR primer pairs for gene expression detection and quantification. Nucleic Acids Res..

[53] Cook, R. D. & Weisberg, S. *Applied regression including computing and graphics*, Vol. 347 (Wiley-Interscience, 1999).

[54] Dean B, Udawela M, Scarr E (2016). Validating reference genes using minimally transformed qpcr data: findings in human cortex and outcomes in schizophrenia. BMC Psychiatry.

