# Supplementary material for: Isoform specific differences in phospholipase C beta 1 expression in the prefrontal cortex in schizophrenia and suicide
Source: NPJ Schizophr. 2017 Apr 19;3:19. doi: 10.1038/s41537-017-0020-x (PMC5441535; doi:10.1038/s41537-017-0020-x)
Supplement: Supplementary file 1 — Supplementary Table S1 [file 41537_2017_20_MOESM1_ESM.doc]

Table S1: Association of PLCB1 levels with potential confounds

|  | | **Cohort 1** | | | | | | | | | | | | | | | **Cohort 2** | | | |
| --- | --- | --- | --- | --- | --- | --- | --- | --- | --- | --- | --- | --- | --- | --- | --- | --- | --- | --- | --- | --- |
|  |  | 1. mRNA | | | | | | | | | | 1. protein | | | | | protein | | | |
|  |  | Age (y) | | PMI (h) | | | Brain pH | DOI (y) | | AP dose (mg)^†^ | RIN | Age (y) | PMI (h) | Brain pH | DOI (y) | AP dose (mg)^†^ | Age (y) | PMI (h) | Brain pH | DOI (y) |
|  | | BA46 | | | | | | | | | | | | | | | BA9 | | | |
| PLCB1a | r^2^  *p* | <0.01  0.64 | 0.01  0.34 | | 0.07  0.03 | | | 0.03  0.25 | 0.03  0.30 | | 0.01  0.40 | 0.02  0.26 | 0.03  0.87 | <0.01  0.76 | <0.01  0.90 | 0.10  0.07 | 0.03  0.17 | <0.01  0.62 | 0.14  <0.01 | 0.02  0.53 |
| PLCB1b | r^2^  *p* | <0.01  0.66 | <0.01  0.78 | | 0.08  0.02 | | | 0.05  0.15 | 0.07  0.09 | | 0.02  0.32 | <0.01  0.60 | 0.01  0.40 | <0.01  0.51 | 0.01  0.64 | 0.09  0.09 | <0.01  0.80 | <0.01  0.65 | 0.03  0.23 | 0.03  0.30 |
|  | | BA9 | | | | | | | | | | | | | | | BA24 | | | |
| PLCB1a | r^2^  *p* | 0.02  0.26 | <0.01  0.56 | | 0.18  <0.01 | | | 0.10  0.03 | <0.01  0.74 | | 0.15  <0.01 | 0.07  0.05 | 0.02  0.33 | 0.01  0.37 | 0.02  0.35 | 0.09  0.08 | <0.01  0.51 | <0.01  0.78 | <0.01  0.98 | <0.01  0.63 |
| PLCB1b | r^2^  *p* | 0.10  <0.01 | <0.01  0.63 | | | 0.05  0.06 | | 0.16  0.01 | 0.01  0.93 | | 0.04  <0.01 | <0.01  0.97 | <0.01  0.82 | <0.01  0.53 | <0.01  0.66 | 0.19  <0.01 | 0.03  0.20 | <0.01  0.81 | <0.01  0.84 | <0.01  0.87 |

^†^Chlorpromazine equivalents; AP, antipsychotic drug; PMI, post-mortem interval; DOI, duration of illness
