# Supplementary material for: Isoform specific differences in phospholipase C beta 1 expression in the prefrontal cortex in schizophrenia and suicide
Source: NPJ Schizophr. 2017 Apr 19;3:19. doi: 10.1038/s41537-017-0020-x (PMC5441535; doi:10.1038/s41537-017-0020-x)
Supplement: Supplementary file 2 — Supplementary Figure S1 [file 41537_2017_20_MOESM2_ESM.doc]

**Cohort 1 Cohort 2**

**Figure S1**: Correlations between experimental measures and subject demographics that showed *p*<0.05. AP, antipsychotic drug; PMI, post-mortem interval; DOI, duration of illness
