# Supplementary material for: Isoform specific differences in phospholipase C beta 1 expression in the prefrontal cortex in schizophrenia and suicide
Source: NPJ Schizophr. 2017 Apr 19;3:19. doi: 10.1038/s41537-017-0020-x (PMC5441535; doi:10.1038/s41537-017-0020-x)
Supplement: Supplementary file 3 — Supplementary Table S2 [file 41537_2017_20_MOESM3_ESM.doc]

Table S2: Analysis of experimental data by suicide status within diagnoses.

| **Cohort 1** | | Schizophrenia suicide  (Cohort 1a n=16, cohort 1b n=12 | | Schizophrenia non-suicide  (Cohort 1a n=28, cohort 1b n=26) | | Comparison (Mann-Whitney test) | |
| --- | --- | --- | --- | --- | --- | --- | --- |
|  |  | Median | IQR | Median | IQR | U | *p* |
| BA9 | mRNA a | 4.17 | 3.28-4.99 | 2.89 | 2.15-3.83 | 121 | 0.01 |
|  | mRNA b | 0.30 | 0.27-0.38 | 0.20 | 0.16-0.29 | 109 | 0.004 |
|  | Protein a | 1.46 | 0.86-1.85 | 1.39 | 1.23-1.56 | 142 | 0.67 |
|  | Protein b | 0.91 | 0.52-1.43 | 1.10 | 0.85-1.39 | 108 | 0.13 |
| **Cohort 2** | | MDD suicide (n=13) | | MDD non-suicide (n=2) | | Comparison (Mann-Whitney test) | |
|  |  | Median | IQR | Median | IQR | U | *p* |
| BA9 | Protein a | 2.67 | 2.60-3.74 | 2.69 | 2.38-3.01 | 10 | 0.59 |
|  | Protein b | 1.74 | 1.48-2.29 | 2.00 | 1.71-2.29 | 10 | 0.69 |
| BA24 | Protein a | 0.97 | 0.75-1.68 | 1.26 | 1.07-1.45 | 11 | 0.80 |
|  | Protein b | 0.90 | 0.54-1.35 | 0.99 | 0.66-1.31 | 12 | 0.93 |
|  |  | BD suicide (n=5) | | BD non-suicide (n=10) | | Comparison (Mann-Whitney test) | |
|  |  | Median | IQR | Median | IQR | U | *p* |
| BA9 | Protein a | 2.62 | 1.61-4.35 | 2.41 | 1.59-2.84 | 20 | 0.59 |
|  | Protein b | 1.83 | 0.97-2.93 | 1.66 | 1.48-1.95 | 25 | 1.00 |
| BA24 | Protein a | 0.99 | 0.82-1.13 | 0.87 | 0.55-1.27 | 22 | 0.76 |
|  | Protein b | 0.82 | 0.67-0.97 | 0.77 | 0.49-0.99 | 21 | 0.68 |

MDD, Major depressive disorder; BD, Bipolar disorder; IQR, inter-quartile range
