# Supplementary material for: Isoform specific differences in phospholipase C beta 1 expression in the prefrontal cortex in schizophrenia and suicide
Source: NPJ Schizophr. 2017 Apr 19;3:19. doi: 10.1038/s41537-017-0020-x (PMC5441535; doi:10.1038/s41537-017-0020-x)
Supplement: Supplementary file 4 — Supplementary Table S3 [file 41537_2017_20_MOESM4_ESM.doc]

Table S3: Demographic data on all subjects

| **Cohort 1** | | | | | | | | | | | | | | |
| --- | --- | --- | --- | --- | --- | --- | --- | --- | --- | --- | --- | --- | --- | --- |
|  | Age (y) | PMI (h) | Brain pH | Sex | Suicide | DOI (y) | Cause of death | AP dose (mg)^†^ | lifetime exposure (g)^†^ | Anti-Ch use | BZP use | RIN  BA9 | RIN  BA46 | ancestry |
| Control | | | | | | | | | | | | | | |
| C1 | 36 | 42 | 6.46 | M | no | N\A | Crush accident | 0 | 0 | N\A | N\A | 8.9 | 7 | CHB |
| C2 | 52 | 22 | 5.98 | M | no | N\A | Pulmonary thromboembolism | 0 | 0 | N\A | N\A | 5.5 | N/D | CEU |
| C3 | 21 | 58 | 6.03 | F | no | N\A | Myocarditis | 0 | 0 | N\A | N\A | 8.6 | 8.4 | CHB |
| C4 | 25 | 50 | 6.48 | M | no | N\A | Exsanguination | 0 | 0 | N\A | N\A | 7.6 | 7 | CEU |
| C5 | 53 | 44.5 | 6.56 | M | no | N\A | Ischaemic heart disease | 0 | 0 | N\A | N\A | 9 | N/D | CEU |
| C6 | 68 | 41 | 6.06 | M | no | N\A | Aortic stenosis | 0 | 0 | N\A | N\A | 9.3 | 8.1 | CEU |
| C7 | 43 | 51 | 6.43 | M | no | N\A | Coronary artery atheroma | 0 | 0 | N\A | N\A | 9.1 | N/D | CEU |
| C8 | 72 | 39 | 6.21 | M | no | N\A | Coronary artery atheroma | 0 | 0 | N\A | N\A | 8.5 | 6.8 | CEU |
| C9 | 53 | 12 | 6.34 | M | no | N\A | Pulmonary thromboembolism | 0 | 0 | N\A | N\A | 8.8 | 8.2 | CHB |
| C10 | 68 | 69 | 6.59 | M | no | N\A | Coronary artery atheroma | 0 | 0 | N\A | N\A | 8.6 | 7.1 | CEU |
| C11 | 68 | 38 | 6.32 | F | no | N\A | Acute asthma | 0 | 0 | N\A | N\A | 7.4 | 6.8 | CEU |
| C12 | 22 | 51 | 6.58 | M | no | N\A | Exsanguination | 0 | 0 | N\A | N\A | 8.6 | 8.3 | CEU |
| C13 | 66 | 43 | 6.37 | F | no | N\A | Acute myocardial infarction | 0 | 0 | N\A | N\A | 9 | 8.4 | CEU |
| C14 | 42 | 63 | 6.34 | M | no | N\A | Cardiomegaly | 0 | 0 | N\A | N\A | 8.4 | 7.9 | CHB |
| C15 | 50 | 65 | 6.4 | M | no | N\A | Ischaemic heart disease | 0 | 0 | N\A | N\A | 8.8 | N/D | CEU |
| C16 | 21 | 40 | 5.82 | M | no | N\A | Acute Epiglottitis | 0 | 0 | N\A | N\A | 8.7 | 7 | CEU |
| C17 | 42 | 26 | 6.32 | M | no | N\A | Coronary artery atheroma | 0 | 0 | N\A | N\A | 8.3 | N/D | CEU |
| C18 | 26 | 24 | 6.42 | M | no | N\A | Electrocution | 0 | 0 | N\A | N\A | 9.3 | 8.8 | CEU |
| C19 | 39 | 52 | 6.26 | F | no | N\A | Mitral valve prolapse | 0 | 0 | N\A | N\A | 7.5 | 8.5 | CEU |
| C20 | 48 | 24 | 6.37 | M | no | N\A | Coronary artery atheroma | 0 | 0 | N\A | N\A | 8.8 | 6.5 | CEU |
| C21 | 48 | 52 | 5.99 | M | no | N\A | Ischaemic heart disease | 0 | 0 | N\A | N\A | 7.4 | 8 | CEU |
| C22 | 34 | 15.5 | 6.4 | M | no | N\A | Ischaemic heart disease | 0 | 0 | N\A | N\A | 9.2 | 6.7 | CEU |
| C23 | 43 | 45 | 6.25 | M | no | N\A | Drowning | 0 | 0 | N\A | N\A | 9.8 | 8.2 | CEU |
| C24 | 65 | 20.5 | 6.47 | M | no | N\A | Acute myocardial infarction | 0 | 0 | N\A | N\A | 8.5 | 7.9 | CEU |
| C25 | 62 | 66 | 6.5 | M | no | N\A | Acute myocardial infarction | 0 | 0 | N\A | N\A | 7.2 | 6.7 | CEU |
| C26 | 22 | 62 | 6.39 | M | no | N\A | Iatrogenic haemorrhage | 0 | 0 | N\A | N\A | 9.5 | 7.7 | CEU |
| Muscarinic receptor deficit schizophrenia | | | | | | | | | | | | | | |
| SZ1 | 36 | 38 | 6.04 | M | yes | 12 | Drug overdose | 200 | 2.4 | yes | no | 8.6 | 6.2 | CEU |
| SZ2 | 51 | 20 | 5.98 | M | no | 32 | Ischaemic Heart Disease | 2000 | 64.0 | no | yes | 9.5 | 8.8 | CEU |
| SZ3 | 21 | 56 | 6.24 | F | yes | 2 | CO poisoning | N/D | N/D | yes | no | 8.3 | N/D | CEU |
| SZ4 | 25 | 49 | 6.38 | M | yes | 2 | Combined Drug Toxicity | 200 | 0.4 | no | no | 9.3 | 8.5 | CEU |
| SZ5 | 53 | 37 | 5.98 | M | no | 30 | Intestinal Ischaemia | 1700 | 51.0 | yes | no | 9.5 | 7.1 | CEU |
| SZ6 | 67 | 21 | 6.46 | M | no | 36 | Pneumonia | 75 | 2.7 | yes | no | 9.1 | 8.3 | CEU |
| SZ7 | 44 | 32 | 6.28 | M | no | 23 | Ischaemic Heart Disease | 600 | 13.8 | no | no | 9.1 | 8.2 | CEU |
| SZ8 | 71 | 48 | 6.45 | M | no | 53 | Food Aspiration | 150 | 8.0 | yes | yes | 9.5 | 8.5 | CEU |
| SZ9 | 53 | 43 | 6.23 | M | no | 7 | Food Aspiration | 200 | 1.4 | no | no | 9.3 | 6.9 | CEU |
| SZ10 | 69 | 44.5 | 6.38 | M | no | 47 | Ischaemic Heart Disease | 100 | 4.7 | yes | no | 8.2 | 6.6 | CEU |
| SZ11 | 68 | 42 | 5.73 | F | no | 40 | Ischaemic heart disease | 400 | 16.0 | yes | no | 8.1 | N/D | CEU |
| SZ12 | 22 | 37 | 6.17 | M | yes | 3 | Combined Drug Toxicity | 200 | 0.6 | no | no | 9.6 | 8.3 | CEU |
| SZ13 | 65 | 50 | 6.35 | F | no | 18 | Ruptured Abdominal Aneurysm | 550 | 9.9 | yes | yes | 8.3 | 8.4 | CEU |
| SZ14 | 41 | 31 | 6.2 | M | yes | 11 | Combined Drug Toxicity | 500 | 5.5 | no | yes | 8.6 | 8.1 | CEU |
| SZ15 | 53 | 9 | 6.29 | M | no | 9 | Coronary artery atheroma | 300 | 2.7 | Yes | yes | 8.8 | 6.5 | CEU/  CHB |
| SZ16 | 19 | 43 | 6.22 | M | yes | 3 | Unascertained | 750 | 2.3 | yes | yes | 8.8 | 6.3 | CEU |
| SZ17 | 42 | 47 | 6.26 | M | no | 22 | Coronary Arterial Atheroma | 1000 | 22.0 | yes | no | 7.5 | 7 | CEU |
| SZ18 | 26 | 52 | 6.39 | M | yes | 2 | CO Poisoning | 500 | 1.0 | yes | yes | 7.7 | 8.9 | CEU |
| SZ19 | 47 | 50 | 6.31 | F | no | 20 | Pneumonia | 600 | 12.0 | no | yes | 8.8 | 7.5 | CEU |
| SZ20 | 48 | 30 | 6.62 | M | no | 24 | Bronchopneumonia | 1250 | 30.0 | no | yes | 8.9 | 9.1 | CEU |

| non- Muscarinic receptor deficit schizophrenia | | | | | | | | | | | | | | |
| --- | --- | --- | --- | --- | --- | --- | --- | --- | --- | --- | --- | --- | --- | --- |
| SZ21 | 38 | 40 | 5.52 | M | no | 15 | Mediastinitis | 169 | 25.3 | yes | no | 5.6 | N/D | CEU |
| SZ22 | 35 | 15 | 6.26 | F | no | 7 | Coronary Arterial Thrombosis | 300 | 2.1 | yes | no | N/D | N/D | CEU |
| SZ23 | 23 | 78 | 6.19 | M | yes | 5 | Multiple Injuries | 300 | 1.5 | yes | no | 9 | 6.9 | CEU |
| SZ24 | 55 | 25 | 6.1 | M | no | 33 | Coronary Arterial Atheroma | 400 | 13.2 | yes | yes | 9.6 | 8.1 | CEU |
| SZ25 | 66 | 39.5 | 6.49 | M | no | 45 | Bronchopneumonia | 1200 | 54.0 | no | yes | 9.3 | 6.2 | CEU |
| SZ26 | 47 | 32.5 | 6.41 | M | no | 27 | Ischaemic Heart Disease | 530 | 14.3 | no | no | 8.8 | 8.5 | CEU |
| SZ27 | 70 | 46 | 5.8 | M | no | 20 | Bronchopneumonia | N/D | N/D | no | no | 9.1 | N/D | CEU |
| SZ28 | 56 | 42 | 6.17 | M | no | 11 | Cancer | 1700 | 18.7 | no | no | 5.6 | N/D | CEU |
| SZ29 | 65 | 42 | 6.29 | M | no | 36 | Bronchopneumonia | 460 | 16.6 | yes | no | 8.4 | 8.7 | CEU |
| SZ30 | 71 | 36 | 5.84 | F | no | 48 | Chronic Obstructive Airways Disease | N/D | N/D | no | no | 5.6 | N/D | CEU |
| SZ31 | 22 | 41.5 | 6.06 | M | yes | 4 | CO poisoning | 1900 | 7.6 | yes | no | 4.8 | N/D | CEU |
| SZ32 | 72 | 58.8 | 6.48 | F | no | 37 | Aspiration Pneumonia | 25 | 0.9 | no | no | 8.5 | 6.8 | CEU |
| SZ33 | 42 | 47 | 6.44 | M | yes | 8 | Hanging | 128 | 1.0 | yes | yes | 6.2 | N/D | CEU |
| SZ34 | 22 | 37 | 6.03 | M | no | 3 | Pericarditis | 450 | 1.4 | no | no | 4.8 | N/D | CEU |
| SZ35 | 38 | 50 | 6.02 | M | no | 4 | Meningoencephalitis | 100 | 0.4 | yes | no | 8.8 | 8.3 | CEU |
| SZ36 | 27 | 22 | 6.28 | M | yes | 8 | Burning | 1200 | 9.6 | no | yes | 8.4 | 6.9 | CEU |
| SZ37 | 48 | 52.5 | 6.21 | F | no | 22 | Pulmonary Thromboembolism | 700 | 15.4 | no | no | N/D | N/D | CEU |
| SZ38 | 47 | 41.5 | 6.52 | M | yes | 21 | Multiple Injuries | 1400 | 29.4 | no | yes | 9 | 9.3 | CEU |
| SZ39 | 46 | 42 | 5.53 | M | no | 12 | Ischaemic heart disease | 160 | 1.92 | no | no | 3.6 | N/D | CEU |
| SZ40 | 65 | 41 | 6.57 | M | no | 35 | Ischaemic heart disease | 150 | 5.25 | no | no | 5.3 | 6.1 | CEU |
| SZ41 | 22 | 48.5 | 6.29 | M | yes | 2 | Asphyxia (plastic bag) | 2920 | 5.84 | no | yes | 6.6 | 6.4 | CEU |
| SZ42 | 42 | 34.5 | 6.26 | M | yes | 15 | Drowning | 610 | 9.15 | no | yes | 8.2 | N/D | CEU |
| SZ43 | 32 | 17 | 6.05 | M | yes | 15 | Carbon monoxide poisoning | 670 | 10.05 | yes | yes | 4.2 | 9.1 | CEU |
| SZ44 | 45 | 68 | 6.48 | M | yes | 12 | Hanging | 300 | 3.6 | no | no | 6.3 | 8.6 | CEU |

| **Cohort 2** | | | | | | | | | | | | |
| --- | --- | --- | --- | --- | --- | --- | --- | --- | --- | --- | --- | --- |
|  | Age (y) | PMI (h) | Brain pH | Sex | Suicide | DOI (y) | Cause of death | AP dose (mg)^†^ | lifetime exposure (mg)^†^ | Anti-Ch use | BZP use | ancestry |
| Control | | | | | | | | | | | | |
| C27 | 42 | 30.5 | 6.45 | M | no | N/A | Ischaemic Heart Disease | 0 | 0 | N/A | N/A | CEU |
| C28 | 52 | 33.75 | 6.52 | M | no | N/A | Ischaemic Heart Disease | 0 | 0 | N/A | N/A | CEU |
| C29 | 47 | 24 | 5.89 | F | no | N/A | Pulmonary Embolism | 0 | 0 | N/A | N/A | CEU |
| C30 | 75 | 53 | 6.01 | F | no | N/A | Multiple Organ Failure | 0 | 0 | N/A | N/A | CEU |
| C31 | 70 | 59 | 6.11 | M | no | N/A | Ischaemic Heart Disease | 0 | 0 | N/A | N/A | CEU |
| C32 | 75 | 69.4 | 6.19 | M | no | N/A | Cardiogenic Shock | 0 | 0 | N/A | N/A | CEU |
| C33 | 55 | 30.5 | 6.69 | M | no | N/A | Coronary Artery Atherosclerosis | 0 | 0 | N/A | N/A | CEU |
| C34 | 66 | 71.75 | 6.47 | M | no | N/A | Coronary Artery Atheroma | 0 | 0 | N/A | N/A | CEU |
| C35 | 80 | 55 | 6.28 | F | no | N/A | Ischaemic Heart Disease | 0 | 0 | N/A | N/A | CHB |
| C36 | 67 | 49.25 | 6.44 | F | no | N/A | Ruptured infrarenal atherosclerotic aortic aneurysm | 0 | 0 | N/A | N/A | CEU |
| C37 | 56 | 24 | 5.88 | F | no | N/A | Pericardial Tamponade | 0 | 0 | N/A | N/A | CEU |
| C38 | 32 | 56 | 6.16 | F | no | N/A | Coronary Artery Atheroma | 0 | 0 | N/A | N/A | CEU |
| C39 | 65 | 36.5 | 6.12 | M | no | N/A | Pulmonary Thromboembolism | 0 | 0 | N/A | N/A | CEU |
| C40 | 62 | 40 | 6.45 | F | no | N/A | Ischaemic Heart Disease | 0 | 0 | N/A | N/A | CEU |
| Major depressive disorder | | | | | | | | | | | | |
| MDD1 | 37 | 57.75 | 6.84 | M | yes | 14 | Hanging | 0 | 0 | no | no | CEU |
| MDD2 | 51 | 41 | 6.71 | M | yes | 15 | Hanging | 0 | 0 | no | no | CEU |
| MDD3 | 50 | 50.5 | 6.85 | F | yes | 40 | Mixed drug toxicity | 0 | 0 | no | no | CEU |
| MDD4 | 77 | 16.7 | 6.49 | F | yes | 26 | Drug Toxicity | 0 | 0 | no | no | CEU |
| MDD5 | 69 | 44.5 | 6.45 | M | yes | 20 | Drowning | 0 | 0 | no | no | CEU |
| MDD6 | 79 | 24 | 6.32 | M | yes | 17 | CO poisoning | 0 | 0 | yes | no | CEU |
| MDD7 | 55 | 47.75 | 6.6 | M | yes | 3 | Hanging | 0 | 0 | no | no | CEU |
| MDD8 | 68 | 60.75 | 6.65 | M | yes | 9 | Hanging | 0 | 0 | no | yes | CEU |
| MDD9 | 87 | 24.5 | 6.44 | F | no | 7 | Chest Infection | 0 | 0 | no | no | CEU |
| MDD10 | 51 | 23.5 | 6.49 | M | no | 25 | Drug Toxicity | 0 | 0 | no | no | CEU |
| MDD11 | 58 | 49 | 6.44 | F | no | 17 | Pulmonary Thromboembolism | 0 | 0 | no | no | CEU |
| MDD12 | 53 | 63 | 6.46 | F | yes | 20-30 | Hanging | 0 | 0 | no | no | CEU |
| MDD13 | 27 | 45 | 6.55 | F | yes | 0.5 | Hanging | 0 | 0 | no | yes | CEU |
| MDD14 | 74 | 46 | 6.64 | M | yes | 15 | Hanging | 0 | 0 | no | no | CEU |
| MDD15 | 54 | 67 | 6.51 | F | yes | 20 | Hanging | 0 | 0 | no | no | CEU |
| Bipolar disorder | | | | | | | | | | | | |
| BP1 | 38 | 24 | 6.42 | M | yes | 3 | CO Poisoning | 300 | 0.9 | no | no | CEU |
| BP2 | 59 | 34 | 6.46 | M | no | 24 | Ruptured Aorta | 0 | 0 | no | no | CEU |
| BP3 | 42 | 25 | 6.54 | F | yes | 20 | Hanging | 0 | 0 | no | yes | CEU |
| BP4 | 64 | 26 | 6.46 | F | no | 8 | Ischaemic Heart Disease | 0 | 0 | N/A | N/A | CEU |
| BP5 | 66 | 17 | 6.41 | M | no | 12 | Food Aspiration | 166 | 2 | N/A | N/A | CEU |
| BP6 | 79 | 8.25 | 6.09 | M | no | 17 | Cholecystitis, Cardiomegaly | 67.5 | 14.8 | N/A | N/A | CEU |
| BP7 | 59 | 37.5 | 5.97 | M | no | 8 | Ischaemic Heart Disease | 0 | 0 | N/A | N/A | CEU |
| BP8 | 61 | 58 | 6.44 | M | no | 40 | Acute Myocardial Infarction | 1500 | 60 | N/A | N/A | CEU |
| BP9 | 74 | 45 | 6.26 | F | no | 35 | Mixed Drug Toxicity | 100 | 3.5 | N/A | N/A | CEU |
| BP10 | 57 | 36 | 6.43 | F | yes | 36 | CO poisoning | 0 | 0 | N/A | N/A | CEU |
| BP11 | 60 | 50 | 6.08 | F | no | 23 | Cardiomegaly | 0 | 0 | N/A | N/A | CEU |
| BP12 | 59 | 41 | 5.68 | F | no | 10 | Ischaemic Heart Disease | 0 | 0 | N/A | N/A | CEU |
| BP13 | 41 | 46.5 | 6.16 | F | yes | 9 | Hypothermia and drug overdose | 648 | 0 | N/A | N/A | CEU |
| BP14 | 77 | 62.5 | 6.06 | M | no | 1.5 | Food Aspiration | 254 | 0 | N/A | N/A | CEU |
| BP15 | 55 | 52 | 6.46 | F | no | 14 | Unascertained | 300 | 4.2 | N/A | N/A | CEU |
| Suicide with no history of psychiatric illness | | | | | | | | | | | | |
| S1 | 45 | 18 | 6.42 | M | yes | N/A | Hanging | N/A | N/A | N/A | N/A | CEU |
| S2 | 52 | 47 | 5.94 | M | yes | N/A | Hanging | N/A | N/A | N/A | N/A | CEU |
| S3 | 60 | 22 | 6.47 | F | yes | N/A | Hanging | N/A | N/A | N/A | N/A | CEU |
| S4 | 63 | 20 | 6.32 | M | yes | N/A | Hanging | N/A | N/A | N/A | N/A | CEU |
| S5 | 51 | 48 | 6.59 | M | yes | N/A | Gunshot | N/A | N/A | N/A | N/A | CEU |
| S6 | 54 | 32.5 | 6.55 | M | yes | N/A | Gunshot | N/A | N/A | N/A | N/A | CEU |
| S7 | 56 | 43.75 | 6.44 | M | yes | N/A | Carbon monoxide poisoning | N/A | N/A | N/A | N/A | CEU |
| S8 | 31 | 40.5 | 6.78 | F | yes | N/A | Hanging | N/A | N/A | N/A | N/A | CEU |
| S9 | 55 | 58.0 | 6.56 | M | yes | N/A | Hanging | N/A | N/A | N/A | N/A | CEU |

^†^Chlorpromazine equivalents; AP, antipsychotic drug; BZB, benzodiazepine; CEU, Western European ancestry; CHB, Han Chinese ancestry; Ch; cholinergic; DOI, duration of illness; N/A, not applicable; N/D, not determined; PMI, post-mortem interval.
